# Supplementary material for: The Nurses’ Innovative Behavior Inventory (NIBI): A development and validation study
Source: PLoS One. 2025 Dec 18;20(12):e0338696. doi: 10.1371/journal.pone.0338696 (PMC12714205; doi:10.1371/journal.pone.0338696)
Supplement: S1 File — (DOCX) [file pone.0338696.s001.docx]

| **ردیف** | **گویه‌ها ‌** | **همیشه** | **اغلب اوقات** | **بعضی اوقات** | **به ندرت** | **هرگز** |
| --- | --- | --- | --- | --- | --- | --- |
| 1 | از کشف ایده‌ها و مفاهیم جدید در پرستاری لذت می‌برم. |  |  |  |  |  |
| 2 | با بیمار و خانواده او ارتباط موثر برقرار می‌کنم. |  |  |  |  |  |
| 3 | علاقمندم تا مسائل پرستاری را به روشی نوآورانه حل کنم. |  |  |  |  |  |
| 4 | توانایی متقاعد کردن سرپرستار و همکاران را برای پذیرش ایده نوآورانه‌ خود دارم. |  |  |  |  |  |
| 5 | دانش و مهارت‌های خود را از طریق یادگیری تکنیک‌ها و روش‌های جدید افزایش می‌دهم. |  |  |  |  |  |
| 6 | برای افزایش مهارت‌های پرستاری خود، به دنبال یادگیری از منابع آموزشی آنلاین نیز هستم. |  |  |  |  |  |
| 7 | براي ایجاد خلاقیت موثر در انجام امور پرستاری، توانمندی و مهارت‌های غیرپرستاری مورد نیازم را نیز فرا می‌گیرم. |  |  |  |  |  |
| 8 | به دنبال شناسایی مشکلات بالقوه یا ناکارآمدی‌ها در پرستاری هستم. |  |  |  |  |  |
| 9 | به چالش‌های نوظهور در مراقبت از بیمار و سیستم‌های مراقبت‌ سلامت توجه دارم. |  |  |  |  |  |
| 10 | از مطالعات علمی موجود برای تولید ایده جدید در مراقبت از بیمار استفاده می‌کنم. |  |  |  |  |  |
| 11 | وقتی با موقعیت‌های پیش‌بینی نشده (مانند انواع خطا در مراقبت از بیمار) مواجه می‌شوم، به دلیل بروز آن فکر می‌کنم. |  |  |  |  |  |
| 12 | مزایا و خطرات احتمالی ایده جدید را قبل از اجرای آن برای بیماران در نظر می‌‌گیرم. |  |  |  |  |  |
| 13 | هنگام معرفی ایده خود، تاکید می‌کنم که چگونه این نوآوری می‌تواند به مراقبت بهتر از بیمار کمک کند. |  |  |  |  |  |
| 14 | در هنگام پیشنهاد و اجرای ایده جدید، محدوده اختیارات خود را به عنوان یک پرستار در نظر می‌گیرم. |  |  |  |  |  |
| 15 | وقتی اجرای ایده نیاز به تأیید یا مجوز دارد، از افراد مطلع راهنمایی می‌خواهم. |  |  |  |  |  |
| 16 | هنگام ارائه ایده‌های نوآورانه از استانداردها و مقررات اخلاقی پیروی می‌کنم. |  |  |  |  |  |
| 17 | برای اجرای ایده خود، به دنبال ترغیب و جلب مشارکت سایر پرستاران هستم. |  |  |  |  |  |
| 18 | برای اجرای ایده خود، به دنبال ترغیب و جلب مشارکت پزشکان و دیگر اعضای تیم درمان هستم. |  |  |  |  |  |
| 19 | خانواده و مراقبان بیمار را در اجرای ایده نوآورانه خود مشارکت می‌دهم. |  |  |  |  |  |
| 20 | ایده مراقبتی خود را ابتدا برای تعداد محدودی از بیماران اجرا می‌کنم. |  |  |  |  |  |
| 21 | برای جلب حمایت اولیه، یک مدل ساده از نوآوری خود را اجرا می‌کنم. |  |  |  |  |  |
| 22 | برای اصلاح ایده مراقبتی خود، از بازخورد بیماران استفاده می‌کنم. |  |  |  |  |  |
| 23 | در جشنواره‌ها یا رویدادها شرکت می‌کنم تا نوآوری خود را به نمایش بگذارم و تبلیغ کنم. |  |  |  |  |  |
| 24 | برای انتشار و به اشتراک‌گذاری اطلاعات در مورد نوآوری خود با دانشگاه‌ها همکاری می‌کنم. |  |  |  |  |  |
| 25 | جزئیات مربوط به نوآوری پرستاری خود را با همکاران، از جمله سایر پرستاران و پزشکان به اشتراک می‌گذارم تا در صورت لزوم در جاهای دیگر اجرا شود. |  |  |  |  |  |
| 26 | وقتی با موانع پیش‌بینی‌نشده در مراقبت از بیمار مواجه می‌شوم، راه‌حل‌های ابتکاری پیدا می‌کنم. |  |  |  |  |  |
| 27 | از ایده‌های موفق سایرین برای بهبود مراقبت از بیمار در بخش خود الهام می‌گیرم. |  |  |  |  |  |
| 28 | از پیشنهادهای نوآورانه بیمار و مراقبین خانوادگی برای رفع نیازهای بیمارم استقبال می کنم. |  |  |  |  |  |
| 29 | برای ارائه يك ایده مراقبتی نوآورانه، نیاز هر مددجو را پیش‌بینی می‌کنم. |  |  |  |  |  |
