# Supplementary material for: The Nurses’ Innovative Behavior Inventory (NIBI): A development and validation study
Source: PLoS One. 2025 Dec 18;20(12):e0338696. doi: 10.1371/journal.pone.0338696 (PMC12714205; doi:10.1371/journal.pone.0338696)
Supplement: S2 File — (DOCX) [file pone.0338696.s002.docx]

1. **Nurses' Innovative Behavior Scale (NIBI)**

| **#** | **Items** | **Always** | **Often** | **Sometimes** | **Rarely** | **Never** |
| --- | --- | --- | --- | --- | --- | --- |
| 1 | I enjoy discovering new ideas and concepts in nursing. | ☐ | ☐ | ☐ | ☐ | ☐ |
| 2 | I establish effective communication with patients and their families. | ☐ | ☐ | ☐ | ☐ | ☐ |
| 3 | I am interested in solving nursing issues innovatively. | ☐ | ☐ | ☐ | ☐ | ☐ |
| 4 | I can persuade my head nurse and colleagues to accept my innovative ideas. | ☐ | ☐ | ☐ | ☐ | ☐ |
| 5 | I enhance my knowledge and skills by learning new techniques and methods. | ☐ | ☐ | ☐ | ☐ | ☐ |
| 6 | I seek to improve my nursing skills through online educational resources. | ☐ | ☐ | ☐ | ☐ | ☐ |
| 7 | To foster creativity in nursing tasks, I acquire non-nursing skills as needed. | ☐ | ☐ | ☐ | ☐ | ☐ |
| 8 | I actively identify potential problems or inefficiencies in nursing practice. | ☐ | ☐ | ☐ | ☐ | ☐ |
| 9 | I pay attention to emerging challenges in patient care and healthcare systems. | ☐ | ☐ | ☐ | ☐ | ☐ |
| 10 | I utilize existing scientific studies to generate new ideas for patient care. | ☐ | ☐ | ☐ | ☐ | ☐ |
| 11 | When I encounter unexpected situations (e.g., errors in patient care), I analyze their causes. | ☐ | ☐ | ☐ | ☐ | ☐ |
| 12 | I consider the potential benefits and risks of a new idea before implementing it for patients. | ☐ | ☐ | ☐ | ☐ | ☐ |
| 13 | When presenting my idea, I emphasize how this innovation can improve patient care. | ☐ | ☐ | ☐ | ☐ | ☐ |
| 14 | I take into account my professional scope of practice as a nurse when proposing and implementing new ideas. | ☐ | ☐ | ☐ | ☐ | ☐ |
| 15 | When an idea requires approval or authorization, I seek guidance from knowledgeable individuals. | ☐ | ☐ | ☐ | ☐ | ☐ |
| 16 | I adhere to ethical standards and regulations when presenting innovative ideas. | ☐ | ☐ | ☐ | ☐ | ☐ |
| 17 | I seek to encourage and engage other nurses in implementing my ideas. | ☐ | ☐ | ☐ | ☐ | ☐ |
| 18 | I aim to persuade and involve physicians and other healthcare team members in executing my ideas. | ☐ | ☐ | ☐ | ☐ | ☐ |
| 19 | I involve patients' families and caregivers in the implementation of my innovative ideas. | ☐ | ☐ | ☐ | ☐ | ☐ |
| 20 | I initially implement my care-related ideas with a limited number of patients. | ☐ | ☐ | ☐ | ☐ | ☐ |
| 21 | To gain initial support, I develop a simple model of my innovation. | ☐ | ☐ | ☐ | ☐ | ☐ |
| 22 | I use patient feedback to refine my care-related ideas. | ☐ | ☐ | ☐ | ☐ | ☐ |
| 23 | I participate in festivals or events to showcase and promote my innovations. | ☐ | ☐ | ☐ | ☐ | ☐ |
| 24 | I collaborate with universities to disseminate and share information about my innovations. | ☐ | ☐ | ☐ | ☐ | ☐ |
| 25 | I share details of my nursing innovations with colleagues, including other nurses and physicians, to facilitate their implementation elsewhere if needed. | ☐ | ☐ | ☐ | ☐ | ☐ |
| 26 | When I encounter unforeseen obstacles in patient care, I find creative solutions. | ☐ | ☐ | ☐ | ☐ | ☐ |
| 27 | I draw inspiration from successful ideas of others to enhance patient care in my unit. | ☐ | ☐ | ☐ | ☐ | ☐ |
| 28 | I welcome innovative suggestions from patients and family caregivers to meet patients' needs. | ☐ | ☐ | ☐ | ☐ | ☐ |
| 29 | I anticipate each patient's specific needs when developing an innovative care idea. | ☐ | ☐ | ☐ | ☐ | ☐ |
