# Supplementary material for: The Nurses’ Innovative Behavior Inventory (NIBI): A development and validation study
Source: PLoS One. 2025 Dec 18;20(12):e0338696. doi: 10.1371/journal.pone.0338696 (PMC12714205; doi:10.1371/journal.pone.0338696)
Supplement: Attachment — Submitted filename: Response to Reviewers(14Sep2025).docx [file pone.0338696.s005.docx]

***Dear prof. Noushin Kohan,***

On behalf of all co-authors, I would like to sincerely thank you and the reviewers for the constructive and insightful comments provided on our manuscript. We carefully reviewed each point raised and have responded to all comments one by one. The reviewers’ feedback was highly valuable and has significantly improved the quality of our work. We are confident that the revised version is now much stronger than the original submission.

For clarity, we have prepared a detailed response table indicating our replies to each comment along with the corresponding page numbers where revisions were made. In the manuscript file, the changes requested by ***Reviewer 1*** are highlighted in yellow, and those requested by ***Reviewer 2*** are highlighted in grey to facilitate the review process.

We greatly appreciate the time and effort invested by you and the reviewers, and we hope that the revised version meets the journal’s standards for publication.

With kind regards,

Ali asghar Ghods

Corresponding Author on behalf of all co-authors

|  | **Reviewers’ Comments** | **Response** | **Page & Line** |
| --- | --- | --- | --- |
|  | **Reviewer #1**  An interesting manuscript has been submitted. Identifying Nurses’ Innovative Behavior and strengthening these behaviors can lead to nurse satisfaction and increasing the quality of nursing services. The result can be the provision of quality nursing care. | We appreciate the positive feedback from Reviewer #1 | --- |
| 1 | The conceptual framework of the study should be written and the concepts in it should be explained. | We appreciate the reviewer's valuable feedback. To address this comment, we have added a new "Conceptual Framework" section to the manuscript. This section now explains the theoretical foundation of the NIBI and defines each of the five dimensions, Nurses' competencies, generating care ideas, feasibility of care ideas, clinical implementation, and promoting innovation, in detail. This revision directly addresses the reviewer's concern and strengthens the manuscript's theoretical foundation. | 3,4 |
| 2 | What is the reason for choosing the title "Inventory"? Considering that a mixed method study with an exploratory approach was conducted, why was this title chosen? | We thank the reviewer for the question regarding the use of the term "Inventory" in the manuscript title. We believe the term is both appropriate and precise for this study. An inventory is an instrument designed to comprehensively assess a wide range of behaviors, traits, or attributes related to a specific construct [1]. Given that our study is not limited to a single dimension but rather measures five distinct, empirically-derived factors, the term "Inventory" accurately reflects the multi-faceted and comprehensive nature of the tool.  Furthermore, our use of a mixed-methods, exploratory design directly supports this choice. The initial qualitative phase, conducted to explore and identify all the facets of innovative behavior from the ground up, is a foundational step consistent with the principles of exploratory sequential mixed-methods research [2]. This approach, combined with the comprehensive literature review, is a robust and widely-accepted methodology for scale development [3]. The resulting multi-dimensional framework, described in the conceptualization phase, is therefore accurately captured by the term "Inventory," which is commonly used for instruments that assess a broad range of attributes rather than a single construct [4]. We believe the title is both accurate and technically correct. |  |
| 3 | In the first phase - Conceptualization, two qualitative studies were conducted. However, these are not mentioned in the abstract and only the psychometric method of the inventory is stated. | We thank the reviewer for this constructive feedback. ***We have revised the abstract to explicitly mention the conceptualization phase, which included a qualitative study and a comprehensive literature review.*** The abstract now accurately reflects our mixed-methods approach, addressing the reviewer's valuable comment. | 1 |
| 4 | The method of Conceptualization needs further explanation. This step was carried out by conducting two qualitative studies, but the type of qualitative study was not stated and it is not clear how the item pool was completed by conducting this phase. | The reviewer's request for a more detailed explanation of the conceptualization phase is a valid one. The in-depth details of the qualitative study, including its design and findings, are fully described in a separate manuscript that is currently under review.  ***To clarify the methodology for this manuscript, we have revised the "Conceptualization" section to more explicitly explain how the item pool was developed.*** We now state that the conceptualization involved both a conventional qualitative content analysis of semi-structured interviews and a comprehensive literature review. | 5 |
| 5 | Although the psychometric stage of research instruments is carried out in several different and separate steps, it is necessary to mention such things as sample size, type of sampling, method, and expression of the most important results in each step. For example, in determining quantitative face validity, what was the sample size? Or in conducting Exploratory and Confirmatory Factor Analysis, how was the sample size selected and nurses divided into two groups? | ***In response, we have revised the Methods and Results sections to provide detailed information for every stage of validation.*** | 5,  6,  7,  8 |
| 6 | The discussion needs to be rewritten. Considering the existing questionnaires on Innovative Behavior and the process of preparing and psychometrically validating each one, the discussion should be rewritten. | ***We have thoroughly revised the Discussion to make it more analytical and comparative.*** The revised section now: (1) contrasts the NIBI with existing international and Iranian instruments, (2) clarifies the distinction between creativity and innovative behavior in nursing, (3) highlights the novelty of the NIBI in its new dimensions, qualitative grounding, and COSMIN-based validation, and (4) expands on its practical implications for education, professional development, and organizational policy. | 16-18 |
|  | **Reviewer #2** |  |  |
| 1 | …some of the results are reported in the Methods section. | We thank the reviewer for this valuable observation. We understand that the reporting of numerical details in the Methods (e.g., sample sizes for EFA and CFA, response rate, and normality thresholds) may have created the impression of presenting results. To clarify, these values were originally included to demonstrate adherence to methodological standards and sample adequacy requirements. Nevertheless, ***to avoid any ambiguity, we have revised the text as follows:***  ***Procedural details (e.g., normality assessment criteria, sample size requirements for factor analysis) are kept in the Methods section, expressed as standards or thresholds.***  ***Actual study-specific findings (e.g., number of participants recruited, achieved response rate, skewness and kurtosis values) are now clearly reported in the Results section.*** | 10,  14,  15 |
| 2 | …your justification for the inadequacy of creativity assessment tools in nursing is insufficient. Creativity in nursing is not necessarily a culture- or society-specific issue; rather, it is primarily a professional competency, and numerous studies have been conducted on this topic. Standardized and widely accepted measurement tools already exist. | We sincerely thank the reviewer for this insightful comment. We agree that creativity is an important professional competency in nursing and acknowledge the availability of several standardized tools for assessing creativity in general contexts. However, our study is focused on innovative behavior, which goes beyond creativity alone. While creativity is primarily concerned with generating new ideas, innovative behavior encompasses a broader process, including idea validation, clinical implementation, and the promotion of innovations in practice [5]. These additional dimensions are critical in nursing, where innovations must align with ethical standards, patient safety, and interprofessional collaboration.  Existing creativity and innovation instruments (e.g., Scott & Bruce, Kleysen & Street, De Jong & Den Hartog) were developed for general organizational settings and may not adequately capture the unique contextual, ethical, and clinical requirements of nursing practice. Our qualitative phase confirmed this gap and identified nursing-specific dimensions, such as “idea validation” and “promoting innovation,” that are absent in existing measures.  ***To address the reviewer’s concern, we have now clarified this distinction in the Introduction and Discussion sections and emphasized why a nursing-specific measure of innovative behavior is required.*** | 2-3  17 |
| 3 | … this study lacks novelty. Furthermore, validated tools for measuring creativity among nurses are already available in the Iranian nursing community. | We sincerely thank the reviewer for this important observation. We respectfully clarify that the novelty of our study lies not in presenting another general creativity scale, but in the development of a nursing-specific inventory of innovative behavior that is theoretically grounded and psychometrically evaluated according to COSMIN guidelines. While we acknowledge the existence of creativity and innovation tools, including those used in the Iranian nursing community, these instruments are either adapted from organizational psychology or focus narrowly on creativity alone.  Our study offers novelty in several ways:  Conceptual contribution – The Nurses’ Innovative Behavior Inventory (NIBI) integrates dimensions such as idea validation and promoting innovation, which emerged from our qualitative study and have not been emphasized in existing instruments.  Contextual relevance – The NIBI is derived from the lived experiences of Iranian clinical nurses, ensuring cultural and clinical alignment while maintaining theoretical generalizability.  Methodological rigor – This is, to our knowledge, the first instrument in this domain in Iran developed and validated following the full COSMIN checklist, ensuring high methodological quality and applicability for both research and practice.  ***To address the reviewer’s concern, we have revised the Discussion and Conclusion sections to explicitly highlight these points of novelty and to contrast the NIBI with existing instruments used in Iran and internationally.*** | 17,  19 |
| 4 | …the Results section of your study is inadequate. | We sincerely thank the reviewer for this observation. We would like to emphasize that all findings from the study were reported precisely and transparently in the Results section, and we made every effort to present the analyses honestly and without omission. Nevertheless, ***we carefully revised the Results section to improve its clarity and comprehensiveness. In the revised version, we have: Structured the psychometric evaluation results in a clearer step-by-step format, Enhanced the reporting and interpretation of factor analysis outcomes, and Clarified the distribution of scores across demographic subgroups.*** | 10,  14,  15 |

**References:**

1.	DeVellis, R.F. and C.T. Thorpe, *Scale development: Theory and applications*. 2021: Sage publications.

2.	Fetters, M.D., L.A. Curry, and J.W. Creswell, *Achieving integration in mixed methods designs—principles and practices.* Health services research, 2013. **48**(6pt2): p. 2134-2156.

3.	Nagpal, D., I. Kornerup, and M.P. Gibson, *Mixed-method research: a basic understanding.* CODS-Journal of Dentistry, 2021. **12**(1): p. 11-16.

4.	Keszei, A.P., M. Novak, and D.L. Streiner, *Introduction to health measurement scales.* Journal of psychosomatic research, 2010. **68**(4): p. 319-323.

5.	Tang, M., *Creativity and innovation: basic concepts and approaches*, in *Handbook of the management of creativity and innovation: Theory and practice*. 2017, World Scientific. p. 3-32.
